# Privacy-preserving analysis of time-to-event data under nested case-control sampling

**DOI:** 10.1177/09622802231215804

**Published:** 2023-12-13

**Authors:** Lamin Juwara, Yi Archer Yang, Ana M Velly, Paramita Saha-Chaudhuri

**Affiliations:** 1 Quantitative Life Sciences, 5620 McGill University, Montreal, Canada; 2 113635 Lady Davis Institute for Medical Research, Montreal, Quebec, Canada; 3 Department of Mathematics and Statistis, 5620 McGill University, Montreal, Quebec, Canada; 4 Department of Dentistry, 5620 McGill University, Montreal, Quebec, Canada; 5 Biogen Digital Health, Biogen Inc., Cambridge, MA, USA

**Keywords:** Survival analysis, data disclosure, privacy-preserving analysis, specimen pooling

## Abstract

Analyses of distributed data networks of rare diseases are constrained by legitimate privacy and ethical concerns. Analytical centers (e.g. research institutions) are thus confronted with the challenging task of obtaining data from recruiting sites that are often unable or unwilling to share personal records of participants. For time-to-event data, recently popularized disclosure techniques with privacy guarantees (e.g., Differentially Private Generative Adversarial Networks) are generally computationally expensive or inaccessible to applied researchers. To perform the widely used Cox proportional hazards regression, we propose an easy-to-implement privacy-preserving data analysis technique by pooling (i.e. aggregating) individual records of covariates at recruiting sites under the nested case-control sampling framework before sharing the pooled nested case-control subcohort. We show that the pooled hazard ratio estimators, under the pooled nested case-control subsamples from the contributing sites, are maximum likelihood estimators and provide consistent estimates of the individual level full cohort HRs. Furthermore, a sampling technique for generating pseudo-event times for individual subjects that constitute the pooled nested case-control subsamples is proposed. Our method is demonstrated using extensive simulations and analysis of the National Lung Screening Trial data. The utility of our proposed approach is compared to the gold standard (full cohort) and synthetic data generated using classification and regression trees. The proposed pooling technique performs to near-optimal levels comparable to full cohort analysis or synthetic data; the efficiency improves in rare event settings when more controls are matched on during nested case-control subcohort sampling.

## 1. Introduction

Large cohort biomarker studies of rare diseases or expensive-to-recruit studies often require merging datasets collected across multiple study centers or databases. Integrating individual data from contributing centers/nodes is a major challenge due to legitimate privacy and ethical concerns.^1^ Analytical centers thus resort to disclosure control techniques such as intermediate statistics release or synthetic data generation to conduct etiologic studies or make predictions about relevant clinical endpoints.^2–5^ Sampling^6^ and noise perturbation^7^ are also sometimes used.

In recent years, the strengths of proposed privacy-preserving techniques have improved considerably. Traditional attempts at inducing privacy, for example, k-anonymity, were primarily focused on masking quasi-identifiers using de-identification techniques such as generalization and suppression.^8,9^ However, with recent advancements in computational power and our presence in multiple social network databases, the potential risk of re-identification is high.^10^ More refined techniques like t-closeness promise better privacy guarantees but the strength of their effectiveness is largely dependent on the reliability of our assumptions about the intruder.^11^ Techniques motivated from cryptography remain the strongest privacy guarantees; they often require different parties run known learning algorithms on a merged version of the local datasets without revealing individual data. Prediction from the learned model however requires the participation of each unit to implement a private scoring algorithm. Consequently, a major challenge of such techniques is the need for high communication and computational cost.^12^

Although not motivated by data privacy concerns, sample aggregation (referred to as pooling henceforth) was initially proposed as a method to circumvent the challenge of testing individual samples in a limited resource setting during World War II.^13^ The method allowed practitioners to combine samples from multiple individuals for a single test in order to reduce the cost of resources needed to test for syphilis. The technique has seen some advancements in recent years, especially for identifying infectious agents in low prevalence settings^14^ and more recently for testing Covid-19.^15^ Alongside these developments, many variations of pooling have been proposed to infer associations between various clinical outcomes of interest and relevant covariates.^16–18^ These techniques have become particularly appealing for the analysis of high-dimensional data where the strategy is utilized to compensate for limited samples or high biological variation.^19^ Pooling is also sometimes used in the literature to mean combining records from various contributing data sites; however, we restrict our use of the term to mean aggregating individual-level covariate.

Of interest in the current article is the application of pooling to preserve patient privacy during the analysis of time-to-event (or survival time) data in a distributed data network. Survival analysis plays a fundamental role in biomedical research, where survival probabilities are routinely computed to inform clinical decisions.^20,21^ Several approaches have been proposed for protecting patient privacy when dealing with survival time data.^22–25^ For example, O’Keefe and Rubin^22^ introduced a privacy technique based on data suppression (e.g. removal of censored events), smoothing, and data perturbation. Yu et al.^23^ also proposed a method based on affine projections of the Cox model. However, these techniques have been challenging to adapt in practice for several reasons: (i) require sustained communication between the analytical center and data node to train the model, for example, distributed regression techniques, (ii) high computational cost to generate synthetic data at the study node, for example, classification and regression trees (CART) synthetic microdata generation,^4^ PATE-GAN,^26^ Differentially Private Generative Adversarial Network (DPGAN),^27^ etc., and (iii) inaccessible to applied researchers without formal statistical training due to intricacies associated with sampling synthetic data from posterior predictive distributions. While pooling has been adapted to preserve patient privacy when analyzing binary outcome data in multi-center studies,^28^ the framework has not been extended to survival-time data under privacy restrictions.

The objectives of the current study include (i) to propose an aggregation based, easy-to-implement, study design for time-to-event outcome under disclosure limitation, (ii) to estimate the hazard ratio of the postulated Cox proportional hazards (PH) model using the aggregate data from the proposed pooling design, and (iii) to estimate the full-cohort survival curves based on the subset of patients that were included in the pooling design. The rest of the manuscript is organized as follows. In Section 2, we first introduce the pooled study design for time-to-event outcome based on a nested case-control subset of the full cohort. We then introduce the full cohort (individual) and pooled subcohort Cox partial likelihood functions under nested case-control sampling. We show that the individual and pooled likelihoods are of the same form and inference could be carried out using existing analytic tools designed for individual likelihood functions. A sampling technique for generating the survival curve based on the subjects included in the pooled design is proposed, and compared to the full cohort and synthetic data. Section 3 discusses various performance assessment metrics relevant for our setting. We present simulations and a real-life example in Section 4 and close with a discussion in Section 5.

## 2. Methods

**Figure 1.**
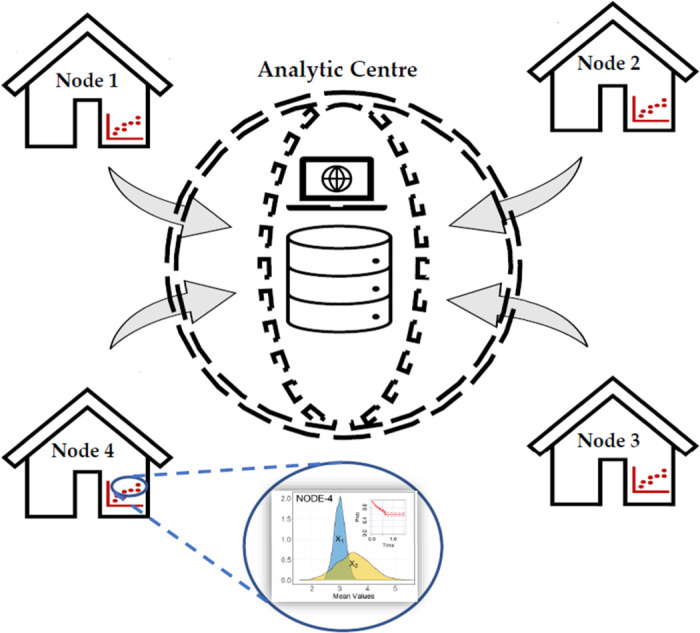
Problem setting—horizontally partitioned time-to-event data between study nodes 1–4. The nodes are only allowed to share aggregate/pooled data with the analytic center. Individual data cannot be shared between nodes or with the center.

Consider a clinical study involving sensitive biomedical data, such as the time-to-death records of HIV/TB co-infected patients, distributed among multiple study centers. Assume that, due to confidentiality agreements and legitimate privacy concerns, the centers are unable to share individual data with analytical centers (see Figure 1 for a schematic representation of the distributed data setting). Our proposed design, based on nested case-control (NCC) subcohort sampling, provides an alternative approach to estimate the hazard ratio (HR) or overall survival curves of time-to-event data while limiting the disclosure of sensitive information from individual-level data. The approach is executed in three stages:

1. NCC design stage: NCC sampling of participants conditional on the event times,
2. Pooling design stage: aggregation/pooling of sampled subcohorts based on event status prior to data sharing, and
3. Estimation stage: estimation of model parameters from the pooled subcohorts and reconstructing approximate event times for pooled cases and controls that constitute the matched sets to estimate overall survival curves.

Steps (1) and (2) are sequentially conducted at each contributing data nodes before the final step (3) is performed at the central analyses node.

### 2.1. Cox PH model

#### Notation and methods

Let N denote the number of participants in a cohort study at risk at baseline t=0 . We denote the time of failure (e.g. disease onset) and time of censoring of subject i by $T_{i}$ and $W_{i}$ , respectively, for i=1,2,…,N . Define the censoring indicator $\delta_{i}=1(T_{i}\leq W_{i})$ and the observed follow-up time $Y_{i}=\text{min}(T_{i},W_{i})$ for the i -th participant. Assume that $T_{i}$ and $W_{i}$ are conditionally independent given the collection of sensitive covariates $Z_{i}=(Z_{i1},Z_{i2},\ldots ,Z_{ip}{)}^{T}$ . The Cox PH model for the covariate-outcome association is given by:

(1) $$\lambda (t|Z_{i})=\lambda_{0}(t)\exp\;(\beta_{1}Z_{i1}+\cdots +\beta_{p}Z_{ip})\equiv \lambda_{0}(t)\exp\;(\beta^{T}Z_{i})$$

where $\beta =(\beta_{1},\ldots ,\beta_{p}{)}^{T}$ , the vector of unknown regression coefficients, are the log HRs characterizing the covariate-outcome association. $\lambda_{0}(t)$ denotes the unspecified baseline hazard.

#### Likelihood for individual-level data

Let the at-risk indicator for subject i be defined as $R_{i}(t)=1(Y_{i}\geq t)$ , the riskset by $R(t)=\{i:Y_{i}\geq t\}$ and the set of individuals surviving past time t as $R(t+)=\{i:Y_{i}>t\}\subset R(t)$ . If we denote the number of subjects that experience an event at t by $n_{t}$ and those surviving past t by $m_{t}$ , then $n_{t}+m_{t}$ subjects make up the riskset R(t) whilst $m_{t}$ subjects make up R(t+) at time t . For the rest of the article, we assume the probability of ties for the continuous survival outcome $Y_{i}$ is negligible (i.e. $n_{t}=1$ ) such that R(t) comprises $m_{t}+1$ subjects. The likelihood of experiencing an event $\delta_{i}=1$ at the observed time $Y_{i}$ is $f_{i}(Y_{i})=S_{i}(Y_{i})\lambda (Y_{i})$ while the contribution from the censored individuals is $S_{i}(Y_{i})$ . Cox (1972) argued that the full likelihood $L_{f}(\beta )=\prod_{i=1}^{N}\lambda (Y_{i}{)}^{\delta_{i}}S_{i}(Y_{i})$ reduces to a partial likelihood under the PH assumption^29^:

(2) $$L_{f}(\beta )\;=\;\prod_{i=1}^{N}[\frac{\exp\;(\beta^{T}Z_{i})}{\sum_{j\in R(t_{i})}\exp\;(\beta^{T}Z_{j})}{]}^{\delta_{i}}$$

where β is estimated by maximizing the partial likelihood. Parameter estimation and inference is readily available via standard statistical programs such as the survival package in R or the PHREG procedure in SAS.

#### 2.1.1. NCC design stage

**Figure 2.**
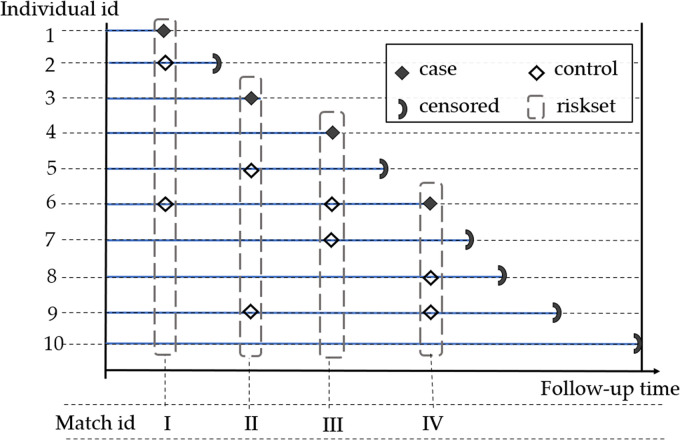
Nested case-control sampling example of two controls per case in a hypothetical cohort of 10 patients. At each event time, two controls are randomly selected in the riskset and matched to the case. Selected controls can become future cases or controls.

NCC subcohort sampling provides an attractive alternative to full cohort analysis, particularly when the analysis is constraint by the amount of available resources^30^ or the amount of information that could be released.^31^ This idea provides a key motivation for the proposed method where aggregate values of NCC subcohorts are utilized to preserve individual records during Cox regression modeling. NCC sampling is also sometimes referred to as riskset sampling or incidence density sampling. A hypothetical example is presented in Figure 2.

In NCC sampling, we embed a case-control study in a cohort such that for all individuals with the outcome of interest (cases), we randomly sample {m:m≥1} controls for each case. In other words, m controls are randomly sampled from the set R(t) at each event time for each case. Denote the set of controls selected from R(t+) by $C_{i}$ and the union of all controls by C , such that $C=\underset{i:\delta_{i}=1}{⋃}C_{i}$ , then the index set of all the subjects included could be expressed as $T=\{i:\delta_{i}=1\}⋃C$ . The sampled riskset of subjects included in the NCC subcohort at each event time is $\tilde{R}(t_{i})=T⋂R(t_{i})$ . Thus, the modified NCC subcohort partial likelihood is now expressed as

(3) $$L_{C}(\beta )\;=\;\prod_{i=1}^{N}[\frac{\exp\;(\beta^{T}Z_{i})}{\sum_{j\in \tilde{R}(t_{i})}\exp\;(\beta^{T}Z_{j})}{]}^{\delta_{i}}$$

which preserves the properties of the full cohort likelihood. Samuelsen^32^ was the first to give a proof for the consistency of the estimates obtained using the NCC partial likelihood in equation (3). As it would be shown later, equation (3) is of the same form as the conditional logistic likelihood for a 1:m matched case-control subcohort.^33,31^ Hence, model parameters and standard errors (SEs) could be estimated with standard statistical programs available for conditional logistic regression, for example, clogit function in the survival package.^34^ The link between the two likelihoods is outlined in Section 2.1.3. Although the above likelihood is useful for estimating the log HRs under resource constraint settings and offers mild privacy protection through the random matching of select controls to cases, individual covariate records and event times still remain unprotected. The next step of the scheme thus involves matched-set pooling to mask individual records.

#### 2.1.2. Pooling design stage: Pooling under NCC sampling

Aggregating individual-level data takes place at the contributing nodes following NCC sampling in stage 1. Upon reducing the Cox partial likelihood in (2) to the NCC subcohort likelihood given in equation (3), the nodes set out to follow the outcome dependent aggregation scheme described by Saha-Chaudhuri and Weinberg^18^ and Saha-Chaudhuri and Juwara.^31^

**Figure 3.**
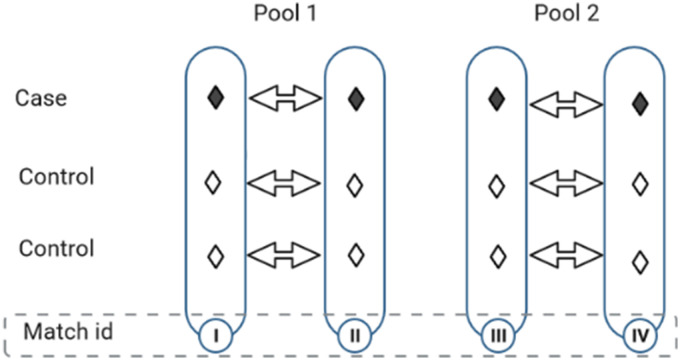
Pooling nested case-control matched sets of participants followed in Figure 2. Each pool is randomly formed from two matched sets by aggregating cases (with cases) and controls (with controls), independently.

For simplicity, we assume individual records of the time-to-event data at each node are sensitive and comprise of individual records of event times, outcome status, exposure $Z_{\left(e\right)}$ , and confounder $Z_{\left(c\right)}$ . Figure 3 demonstrates pooled NCC subcohort sampling on the hypothetical cohort data provided in Figure 2. Covariate values of the matched sets (I vs. II and III vs. IV) are randomly aggregated via averaging while keeping the matching intact. For example, the cases and controls in matched sets I and II are independently pooled in order to maintain the outcome status of the pooled set (i.e. cases are pooled with cases and controls are pooled with controls).

A snippet of the node-level pooled subcohort that is anticipated for release at each node is presented in Table 1. In the example provided, the pooled exposure values of pool id 1 are obtained by adding the covariate values of cases in matched set I with cases in matched set II (i.e. $Z_{\left(e\right)}^{\text{pool}}$ for pooling individuals 1 and 3 = $z_{1}^{\text{I}}+z_{3}^{\text{II}}$ ) and controls with other controls in matched sets I and II, respectively (e.g. $Z_{\left(e\right)}^{\text{pool}}$ for individuals 2 and 5 = $z_{2}^{\text{I}}+z_{5}^{\text{II}}$ ). For the released event time $Y_{i}$ of the pooled cases, only the minimum perturbed event time of the cases pooled is released. The nodes do not release data for cells marked ⊘ , including event times for cell where the riskset size is 5 or less.^35^ A detailed description of disclosed aggregate data and the role they play in recovering some of the information lost to pooling is provided in Section 2.1.4.

**Table 1.** Pooled survival data created at each node under NCC subcohort sampling using the toy data of Figure 2. Information marked ⊘ is not retained during pooling. Only aggregate covariate values are shared with the analysis center. Riskset size ( $|R_{i}(t+)|$ ) is omitted from pooled data if the value is less than 5. The perturbation τ is sampled from $|N(0,(Y_{i+1}-Y_{i})/2)|$ . $Y_{\max}$ is the end of the study.

Time $Y_{i}^{\text{pool}}$	$|R_{i}(t+)|$	Status	$Z_{\left(e\right)}^{\text{ pool}}$	$Z_{\left(c\right)}^{\text{ pool}}$	Pool size κ	Pool id
$\min(Y_{1},Y_{3})+\tau$	9	1	$z_{1}^{\text{I}}+z_{3}^{\text{II}}$	$z_{1}^{\text{I}}+z_{3}^{\text{II}}$	2	1
⊘	⊘	0	$z_{2}^{\text{I}}+z_{5}^{\text{II}}$	$z_{2}^{\text{I}}+z_{5}^{\text{II}}$	2	1
⊘	⊘	0	$z_{6}^{\text{I}}+z_{9}^{\text{II}}$	$z_{6}^{\text{I}}+z_{9}^{\text{II}}$	2	1
$\min(Y_{4},Y_{6})+\tau$	6	1	$z_{4}^{\text{III}}+z_{6}^{\text{IV}}$	$z_{4}^{\text{III}}+z_{6}^{\text{IV}}$	2	2
⊘	⊘	0	$z_{6}^{\text{III}}+z_{8}^{\text{IV}}$	$z_{6}^{\text{III}}+z_{8}^{\text{IV}}$	2	2
⊘	⊘	0	$z_{7}^{\text{III}}+z_{9}^{\text{IV}}$	$z_{7}^{\text{III}}+z_{9}^{\text{IV}}$	2	2
$Y_{\max}$	⊘	⊘	⊘	⊘	⊘	⊘

The proposed pooling design can be extended to include pooled versions of additional confounders. Thus, if variables are available based on individual determinations, for example, by questionnaires, one could form set-based versions by summing the values across the individuals in the set. For categorical data, indicator variables of the categories could be used and pooled accordingly. Similarly, transformations of covariates (e.g. polynomial or log) and effect modifiers can be handled, but need more care.

#### 2.1.3. Estimation stage

Once the pooled NCC subcohorts have been created at the nodes, the next step is to share the aggregate values (as shown in Table 1) with the analytical center where they are combined into a single data file for analyses. We mainly focus on HR estimation under the postulated Cox model and estimation of overall survival curves.

#### HR estimation with pooled NCC likelihood

We now describe the likelihood contribution of individuals pooled at a single node. Consider a 1:m matched case-control sets at any arbitrary node following NCC subcohort sampling, along with measurements of a single exposure and confounder. Denote the outcome event indicators of the i -th matched set by $\left(\delta_{i}^{1},\delta_{i}^{2},\ldots ,\delta_{i}^{m+1}\right)$ and the random variable version by $\left(D_{i}^{1},D_{i}^{2},\ldots ,D_{i}^{m+1}\right)$ , the observed survival times by $\left(Y_{i}^{1},Y_{i}^{2},\ldots ,Y_{i}^{m+1}\right)$ with $Y_{i}^{j}=Y_{i}$ for all j representing the event time of the matched set, the exposure variable by $\left(Z_{i(e)}^{1},Z_{i(e)}^{2},\ldots ,Z_{i(e)}^{m+1}\right)$ , and the confounder by $\left(Z_{i(c)}^{1},Z_{i(c)}^{2},\ldots ,Z_{i(c)}^{m+1}\right)$ for i=1,2,…,n . The time matched samples are now randomly aggregated based on event status. For an arbitrary pool size κ and n matched sets, the pooled data generated is comprised of n/κ matched samples of aggregate covariates and outcome events. The likelihood contribution of the pooled matched set $i^{\prime }\in \{1,2,\ldots ,n/\kappa \}$ is expressed as

$$\begin{array}{rl} \mathrm{Pr} & (D_{i^{\prime }}^{1}=1|\sum_{j=1}^{m+1}\delta_{i^{\prime }}^{j}=1,\{Y_{i^{\prime }}^{j}{\}}_{j=1}^{m+1},\{Z_{i^{\prime }(e)}^{j}{\}}_{j=1}^{m+1},\{Z_{i^{\prime }(c)}^{j}{\}}_{j=1}^{m+1},\theta )\\ & =\;\frac{\mathrm{Pr}(D_{i^{\prime }}^{1}=1,D_{i^{\prime }}^{2}=0,\ldots ,D_{i^{\prime }}^{m+1}=0,\{Y_{i^{\prime }}^{j}{\}}_{j=1}^{m+1}|\{Z_{i^{\prime }(e)}^{j}{\}}_{j=1}^{m+1},\{Z_{i^{\prime }(c)}^{j}{\}}_{j=1}^{m+1},\theta )}{\mathrm{Pr}(D_{i^{\prime }}^{1}+D_{i^{\prime }}^{2}+\cdots +D_{i^{\prime }}^{m+1}=1,\{Y_{i^{\prime }}^{j}{\}}_{j=1}^{m+1}|\{Z_{i^{\prime }(e)}^{j}{\}}_{j=1}^{m+1},\{Z_{i^{\prime }(c)}^{j}{\}}_{j=1}^{m+1},\theta )}\\ & =\;\frac{\mathrm{Pr}(D_{i^{\prime }}^{1}=1,D_{i^{\prime }}^{2}=0,\ldots ,D_{i^{\prime }}^{m+1}=0,\{Y_{i^{\prime }}^{j}{\}}_{j=1}^{m+1}|\{Z_{i^{\prime }(e)}^{j}{\}}_{j=1}^{m+1},\{Z_{i^{\prime }(c)}^{j}{\}}_{j=1}^{m+1},\theta )}{\sum_{D}\mathrm{Pr}(D_{i^{\prime }}^{1}=\delta_{i^{\prime }}^{1},D_{i^{\prime }}^{2}=\delta_{i^{\prime }}^{2},\cdots +D_{i^{\prime }}^{m+1}=\delta_{i^{\prime }}^{m+1},\{Y_{i^{\prime }}^{j}{\}}_{j=1}^{m+1}|\{Z_{i^{\prime }(e)}^{j}{\}}_{j=1}^{m+1},\{Z_{i^{\prime }(c)}^{j}{\}}_{j=1}^{m+1},\theta )}\\ & =\;\frac{\mathrm{Pr}(D_{i^{\prime }}^{1}=1,Y_{i^{\prime }}^{1}|Z_{i^{\prime }(e)}^{1},Z_{i^{\prime }(c)}^{1},\theta )\times \cdots \times \mathrm{Pr}(D_{i^{\prime }}^{m+1}=0,Y_{i^{\prime }}^{m+1}|Z_{i^{\prime }(e)}^{m+1},Z_{i^{\prime }(c)}^{m+1},\theta )}{\sum_{D}\mathrm{Pr}(D_{i^{\prime }}^{1}=\delta_{i^{\prime }}^{1},Y_{i^{\prime }}^{1}|Z_{i^{\prime }(e)}^{1},Z_{i^{\prime }(c)}^{1},\theta )\times \cdots \times \mathrm{Pr}(D_{i^{\prime }}^{\left(m+\right)}=\delta_{i^{\prime }}^{m+1},Y_{i^{\prime }}^{m+1}|Z_{i^{\prime }(e)}^{m+1},Z_{i^{\prime }(c)}^{m+1},\theta )} \end{array}$$

where $Y_{i^{\prime }}^{j}$ represents the minimum observed time of the cases or controls constituting that pool set, $Z_{i^{\prime }(e)}^{j}=\kappa^{-1}\sum_{i=1}^{\kappa }Z_{i(e)}^{j}$ , and $Z_{i^{\prime }(c)}^{j}=\kappa^{-1}\sum_{i=1}^{\kappa }Z_{i(c)}^{j}$ for all j(j=1,…,m+1) .

D represents the set:

$$\left(\delta_{i^{\prime }}^{1},\delta_{i^{\prime }}^{2},\ldots ,\delta_{i^{\prime }}^{m+1})\in \{(1^{\left(1\right)},0^{\left(2\right)},\ldots ,0^{\left(m+1\right)}),(0^{\left(1\right)},1^{\left(2\right)},\ldots ,0^{\left(m+1\right)}),\ldots ,(0^{\left(1\right)},0^{\left(2\right)},\ldots ,1^{\left(m+1\right)})\right\}$$

Assuming a PH model for all $j:\delta_{i^{\prime }}^{j}=0$ , the probabilities give

$$\mathrm{Pr}(D_{i^{\prime }}^{j}=0,Y_{i^{\prime }}^{j}|Z_{i^{\prime }(e)}^{j},Z_{i^{\prime }j}^{\left(c\right)},\theta )\overset{\theta ∼(\lambda_{0i^{\prime },\beta_{1},\beta_{2}})}{\propto }\underset{Q_{i^{\prime }}^{j}(Y_{i^{\prime }})}{\underbrace{\exp\;(-\exp\;(\beta_{1}Z_{i^{\prime }(e)}^{j}+\beta_{2}Z_{i^{\prime }(c)}^{j})\int_{0}^{Y_{i^{\prime }}^{j}}\lambda_{0i^{\prime }}(\tau )d\tau )}}$$

For all $j:\delta_{i^{\prime }}^{j}=1$ ,

$$\mathrm{Pr}(D_{i^{\prime }}^{j}=1,Y_{i^{\prime }}^{j}|Z_{i^{\prime }(e)}^{j},Z_{i^{\prime }(c)}^{j},\theta )\overset{\left(\lambda_{0i^{\prime },\beta_{1},\beta_{2}}\right)}{\propto }\lambda_{0i^{\prime }}(Y_{i^{\prime }}^{j})\exp\;(\beta_{1}Z_{i^{\prime }(e)}^{j}+\beta_{2}Z_{i^{\prime }(c)}^{j})\times Q_{i^{\prime }}^{j}(Y_{i^{\prime }})$$

where $\lambda_{0i^{\prime }}(Y_{i^{\prime }}^{j})$ denotes the baseline hazard specific to the $i^{\prime }$ -th observation of the matched subcohort for $j:\delta_{i^{\prime }}^{j}=1$ and $Q_{i^{\prime }}^{j}(Y_{i^{\prime }})=\exp\;(-\exp\;(\beta_{1}Z_{i^{\prime }(e)}^{j}+\beta_{2}Z_{i^{\prime }(c)}^{j})\int_{0}^{Y_{i^{\prime }}^{j}}\lambda_{0i^{\prime }}(\tau )d\tau )$ represent the survival terms common to the case and control matched sets. We can thus rewrite the likelihood contribution to the pooled matched set as

$$\begin{array}{rl} & \mathrm{Pr}(D_{i^{\prime }}^{1}=1|\sum_{j=1}^{m+1}\delta_{i^{\prime }}^{j}=1,\{Y_{i^{\prime }}^{j}{\}}_{j=1}^{m+1},\{Z_{i^{\prime }(e)}^{j}{\}}_{j=1}^{m+1},\{Z_{i^{\prime }(c)}^{j}{\}}_{j=1}^{m+1},\theta )\\ & =\frac{\lambda_{0i^{\prime }}(Y_{i^{\prime }}^{1})\exp\;(\beta_{1}Z_{i^{\prime }(e)}^{1}+\beta_{2}Z_{i^{\prime }(c)}^{1})Q_{i^{\prime }}^{1}\times Q_{i^{\prime }}^{2}\times \cdots \times \;Q_{i^{\prime }}^{m+1}}{\sum_{D}(\lambda_{0i^{\prime }}(Y_{i^{\prime }}^{1})\exp\;(\beta_{1}Z_{i^{\prime }(e)}^{1}+\beta_{2}Z_{i^{\prime }(c)}^{1}){)}^{\delta_{i^{\prime }}^{1}}Q_{i^{\prime }}^{1}\times \cdots \times (\lambda_{0i^{\prime }}(Y_{i^{\prime }}^{m+1})\exp\;(\beta_{1}Z_{i^{\prime }(e)}^{m+1}+\beta_{2}Z_{i^{\prime }(c)}^{m+1}){)}^{\delta_{i^{\prime }}^{m+1}}Q_{i^{\prime }}^{m+1}}\\ & =\frac{\exp\;(\beta_{1}Z_{i^{\prime }(e)}^{1}+\beta_{2}Z_{i^{\prime }(c)}^{1})}{\exp\;(\beta_{1}Z_{i^{\prime }(e)}^{1}+\beta_{2}Z_{i^{\prime }(c)}^{1})+\exp\;(\beta_{1}Z_{i^{\prime }(e)}^{2}+\beta_{2}Z_{i^{\prime }(c)}^{2})+\cdots +\exp\;(\beta_{1}Z_{i^{\prime }(e)}^{m+1}+\beta_{2}Z_{i^{\prime }(c)}^{m+1})} \end{array}$$

The product of the likelihood contribution $\mathrm{Pr}(D_{i^{\prime }}^{1}=1|\cdot )$ over all the pooled matched sets $i^{\prime }\in \{1,2,\ldots ,n/\kappa \}$ gives the expression

(4) $$\begin{array}{r} L_{\mathrm{Pooled}}(\beta )\;=\;\prod_{i^{\prime }=1}^{n/\kappa }[\frac{\exp\;(\beta_{1}Z_{i^{\prime }(e)}^{1}+\beta_{2}Z_{i^{\prime }(c)}^{1})}{\sum_{j^{\prime }\in R(t_{i^{\prime }})}\exp\;(\beta_{1}Z_{i^{\prime }(e)}^{j^{\prime }}+\beta_{2}Z_{i^{\prime }(c)}^{j^{\prime }})}{]}^{\delta_{i^{\prime }}} \end{array}$$

which results in the same likelihood form as the NCC subcohort likelihood in equation (3) with the same regression parameters. Thus, predictions and inference could be conducted using the pooled data instead of individual level data. The consistency of the pooled logistic likelihood in estimating the parameters of individual level likelihood has been shown by Saha-Chaudhuriet al.^36^ Our derivation closely follows the conditional logistic likelihood derivation of Clayton and Hills^37^ and Langholz and Goldstein^30^ and Langholz and Clayton.^38^

Utilizing the well-established equivalence between likelihood of the NCC subcohort and the likelihood of conditional logistic regression,^39,36^ inference on the MLEs could be carried out by using readily available packages for conditional logistic regression or stratified Cox regression.^34^ The estimated parameters are interpreted as log HRs rather than the traditional log odds ratios derived from conditional logistic likelihood. Moreover, standard inference techniques applicable to conditional logistic likelihood can be employed to estimate the SEs of the pooled subcohort estimators. Of note, the units of analysis for the pooled NCC subcohort are the pools themselves, as opposed to individual measurements.

As mentioned earlier, the AC receives pooled NCC subcohorts (see Table 1) from each contributing node. This includes partial information on the observed event times of the pooled cases, the number of individuals making up each riskset at the node level if it is more than five individuals, the pool event status (1 if cases are pooled and 0 if controls are pooled), and their corresponding aggregate covariate values. While the log HRs associated with the covariates could be estimated using the pooled subcohorts without any need for the matched event times; to estimate the overall survival curves, the individual event times of subjects making up the pools would need to be recovered.

#### 2.1.4. Estimation of survival curves

Suppose we want to reconstruct the overall survival curve S(t)=Pr(Y>t) ( t=0,1,… are the event times) of the full cohort data using the pooled NCC subcohorts that are shared with the analytical center. When full cohort data is available, several estimators could be utilized for calculating the survival function. In biomedical research, the Kaplan–Meier (KM) estimator is usually the preferred method and is given by

(5) $$\hat{S}(t)=\prod_{i:Y_{i}\leq t}(1-\frac{d_{i}}{n_{i}})$$

where $Y_{i}$ represents the time at which one or more events happened, $d_{i}$ is the number of events (e.g. deaths) at $Y_{i}$ , and $n_{i}$ is the number of individuals surviving past the event time. The KM estimator is easy to evaluate when individual-level data is accessible. However, with pooled NCC subcohorts under privacy restrictions, information about individual event time (and censoring time for the controls) is inaccessible to the analyst.

In order to accurately estimate the survival curve based on pooled NCC subcohorts, a procedure for regenerating the observed event/censoring times of the cases and controls making up the pooled matched sets is needed. To perform this reconstruction, the AC has access to the pooled survival times of cases, the size of the riskset at each disclosed event time, and the event status of the pools. Of note, only the minimum event time of the pooled cases from a given pooled matched set is passed on to the AC. The survival time for the rest of the cases making up the pool and all of the controls would need to be re-generated to estimate the survival curves.

Let the pooled event times be ordered such that $Y_{1}^{\text{pool}}<Y_{2}^{\text{pool}}<\cdots <Y_{\kappa }^{\text{pool}}<Y_{\max}$ , the observed survival time of the cases and controls that make up the pooled matched sets are derived using the following algorithm:


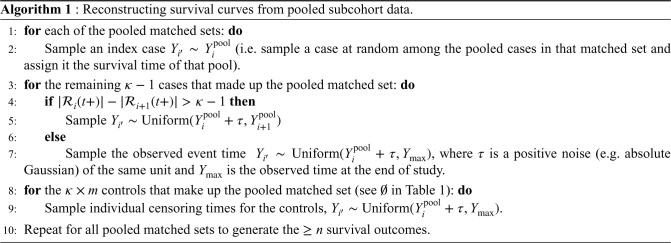


These choices of sampling ensure the ranking of event status (of individuals in the full cohort) are appropriately reconstructed. In other words, the number of individuals that make up each riskset is correctly retrieved. Even though the sampling may not generate precise estimates of observed event times per se, it preserves their ranking and consequently the count of individuals in each riskset. Hence, the estimator in equation (5) could be employed to accurately estimate the survival risk of individuals given a reasonable time window.

**Figure 4.**
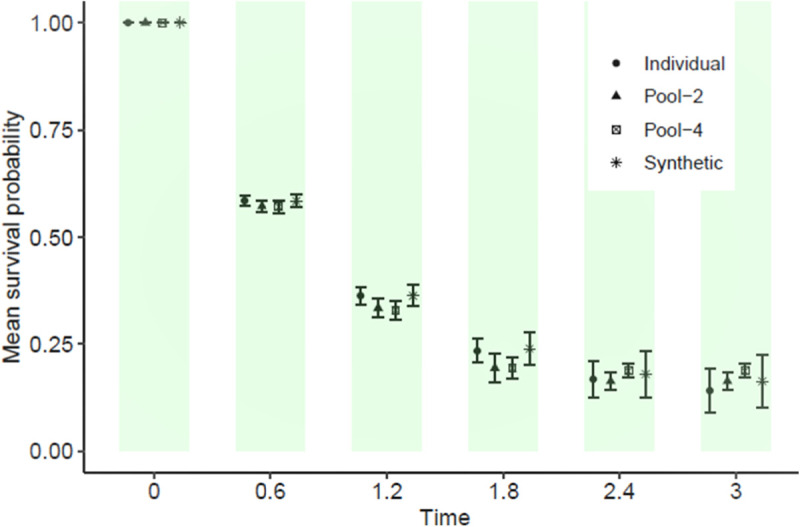
Average estimates of survival probabilities comparing individual, reconstructed pooled subcohorts, and classification and regression trees (CART)-generated synthetic data from 1000 simulated datasets each of size 5000. Mean survival probabilities and error bars are provided.

Of note, the proposed reconstruction generates excess controls resulting from repeated sampling of controls during NCC sampling. Therefore, to ensure a fair comparison of survival curves generated from different pooled subcohorts, we recommend randomly sampling out the excess controls. For example, the pooled toy example of Figure 3 constitutes 12 data points whereas the original full cohort only included 10 individuals. Hence the reconstruction (of individual survival outcomes) will generate event times for two more controls than what we started with which would need to be randomly sampled out to ensure a fair comparison to full cohort data. We demonstrate the performance of the sampling technique for estimating absolute survival risk after time t using simulations in Figure 4.

### 2.2. Alternative approaches for sharing microdata

In recent years, various synthetic data generation methods (e.g. CART synthesis, DP-GAN, etc.) have gained popularity as advancements over traditional anonymization techniques like suppression, aggregation, and noise perturbation.^3,4,26,27^ These methods have emerged as effective approaches to facilitate the sharing of sensitive microdata while preserving data integrity. CART synthetic data generation, in particular, has attracted a lot of interest and is employed by various national agencies for data disclosure, for example, the Scottish Longitudinal Study Development and Support Unit (https://sls.lscs.ac.uk/guides-resources/synthetic-data/). We thus use it as a standard technique for generating synthetic data and compare its performance to the proposed method.

The CART algorithm partitions the predictor space into subsets, using unit partitions, in order to obtain subsets with consistent outcomes.^40,41^ Homogeneity at each split is tracked in CART through the utilization of an impurity function. The best split is determined by thoroughly exploring all variables and split values, ultimately selecting the split that minimizes impurity the most. Examples of such functions include the entropy criterion and the gini index. The optimal size of the CART model is determined by a complexity measure that carefully balances the accuracy of the model and the size of the resulting tree. Initially, the tree is grown to its maximum size and subsequently pruned to refine its structure. We present a tree structure representation of the binary splitting of CART in the Supplemental Appendix.

CART is frequently used for imputing missing categorical variables; however, in the current setting, we are primarily interested in its adoption for synthesizing confidential data. The algorithm is employed to replace individuals at high risk of re-identification with records generated by sampling from the posterior predictive distribution of the outcome fitted with the CART model. In practice, we use the synthpop R package^42^ to generate synthetic copies of the simulated datasets. We assume all the variables are quasi-identifiers that could potentially aid information leakage. A detailed description of the method is given by Reiter.^4^ For the rest of the article, we use the term “synthetic CART” to refer to this mechanism.

## 3. Performance metrics

The performance of the pooled subcohorts, under the postulated Cox PH regression framework, is assessed based on several metrics. Four main performance metrics are considered in assessing the usefulness of the released data. For the estimation of the log HR, we considered: (1) mean absolute error (bias) of the log HRs $\hat{\beta }$ , (2) SE estimates SE( $\hat{\beta }$ ), and (3) relative efficiency (Reff) of the log HRs. To compare the survival curves generated from pooled subcohorts to full cohort data, we considered (4) the mean survival probability and SEs. The first three are particularly relevant for conducting etiological studies while the fourth metric is useful for assessing the suitability of the released data for meaningful endpoint prediction.

1. The mean absolute bias estimate for each log HR ${\hat{\beta }}_{j}$ is expressed as $\mu_{bias}\;({\hat{\beta }}_{j})=\sum_{r=1}^{k}|\beta_{j}-{\hat{\beta }}_{jr}|/k,$ where k is the number of repetitions and ${\hat{\beta }}_{jr}$ is the maximum likelihood estimate (MLE) of the j -th log HR derived from the r -th repetition. This metric is particularly useful since covariate aggregation has been shown to lead to important underestimation of the exposure effect and systemic bias towards the null in Cox PH regression.^43^ We compare the bias associated with the log HRs estimated from full cohort data, pooled NCC subcohorts, and synthetic data.
2. Similar to standard likelihood theory, the variance $V(\hat{\beta })$ can be estimated by inverting the second derivative of the partial likelihood function which is used to derive the SE. In practice, the SE is available from the survival packages.^30^ If the $\text{SE}({\hat{\beta }}_{j})$ of the j -th covariate is estimated over k repetitions, we use the summary $\sum_{r=1}^{k}\text{SE}({\hat{\beta }}_{jr})/k$ .
3. The relative efficiency (Reff) of the log HR estimates compares the median model-based variance of the log HR to the empirical variance computed from log HR estimates of the re-sampled datasets.^44^ This is expressed as $\text{Reff}({\hat{\beta }}_{emp},{\hat{\beta }}_{mod})=V({\hat{\beta }}_{mod})/V({\hat{\beta }}_{emp}),$ where $V({\hat{\beta }}_{emp})$ is the empirical variance and $V({\hat{\beta }}_{mod})$ is the model-based variance, respectively.
4. The survival curves generated from the pooled subcohorts and synthetic data are each compared to the survival curve from the full cohort data. This comparison is based on their empirical mean survival probabilities and SEs obtained from multiple simulated datasets.

## 4. Applications

We used simulations and real data examples to evaluate the performance of the proposed method. The utility of pooled NCC data, under the proposed framework, was assessed using the performance metrics presented in previous sections. Our analysis considered varying combinations of pooled sizes, case-control matched sets, and censoring proportions. To do this, we compared PH regression models for the full cohort (i.e. individual data), pooled NCC subcohorts, and CART-generated synthetic data.

### 4.1. Simulation study

The simulations were based on 1000 repetitions, each with a sample size N=5000 . We assumed the PH model $\lambda (t|Z)=\lambda_{0}(t)\exp\;(\beta_{1}Z_{1}+\beta_{2}Z_{2})$ , with the covariates $Z=(Z_{1},Z_{2}{)}^{T}$ sampled from a bivariate normal distribution. We considered various parameter combinations and present the results for the following setting:

$$\left[\begin{array}{c} Z_{1}\\ Z_{2} \end{array}\right]=N\left(\left[\begin{array}{c} 1.5\\ 2.8 \end{array}\right],\left[\begin{array}{cc} 0.04 & -0.024\\ -0.024 & 0.36 \end{array}\right]\right)$$

where the correlation $\rho_{Z_{1},Z_{2}}=-0.2$ . The regression parameters $\left(\beta_{1},\beta_{2}\right)$ were fixed to (−1.5,0.5) . The true failure times T and censoring times C were generated from Weibull distributions scale parameters $\lambda_{k}\exp\;(-\beta_{1}Z_{1}-\beta_{2}Z_{2})$ and $\lambda_{C}$ , respectively, whereas the shape parameter was fixed to 1 for both times. The observed survival time is taken to be the minimum of T and C . The values of the fixed parameters $\lambda_{k}$ and $\lambda_{C}$ were varied to obtain different observed event or censoring prevalence. For each simulated full cohort, we generated a synthetic copy using CART as previously described. We also created NCC subcohorts for each full cohort by selecting all the cases and choosing m=(2,5,10) controls per case. These subcohorts were then used for the pooling and estimation scheme outlined in Section 2.

For each constructed data type, we estimated the log HRs associated with the covariates, the model-based SE, mean absolute bias (bias), and relative efficiency (Reff), and compared them to the gold standard estimates of the full cohort. Specifically, the metrics were computed for full cohort, pooled NCC subcohorts of pool sizes 2 and 4, and CART-generated synthetic datasets. The empirical coverage (Cov) was also assessed for each data type and compared to the nominal coverage of 0.95. Additionally, the robustness of the subcohorts constructed to varying parameter values were assessed by alternatively fixing one of $\beta_{1}$ or $\beta_{2}$ to zero while varying the other parameter over the range of log HR values (−2, 2).

**Table 2.** Log HR ( $\hat{\beta }$ ) estimates of individual, pooled NCC subcohorts, and CART-generated synthetic data under the Cox PH model assumption. Estimates of SE, mean absolute bias (bias), relative efficiency (Reff), and coverage probability are shown. The pools were formed under 1:5 matched NCC subcohorts. Nominal coverage was 0.95.

		Estimate	SE	Bias	Reff	Coverage
Censoring	=10%
$\beta_{1}$ :	Individual data	−1.50	0.15	0.12	1.13	0.95
	Pool-2 subcohort	−1.51	0.17	0.13	1.06	0.97
	Pool-4 subcohort	−1.50	0.19	0.15	0.94	0.94
	Synthetic data	−1.47	0.15	0.18	0.65	0.85
$\beta_{2}$ :	Individual data	0.50	0.05	0.04	0.79	0.96
	Pool-2 subcohort	0.50	0.05	0.05	0.89	0.95
	Pool-4 subcohort	0.49	0.06	0.05	0.98	0.97
	Synthetic data	0.50	0.05	0.06	0.68	0.86
Censoring	=30%
$\beta_{1}$ :	Individual data	−1.50	0.14	0.12	0.88	0.95
	Pool-2 subcohort	−1.52	0.16	0.13	0.89	0.93
	Pool-4 subcohort	−1.51	0.17	0.15	0.84	0.89
	Synthetic	−1.50	0.14	0.16	0.71	0.81
$\beta_{2}$ :	Individual data	0.49	0.05	0.04	0.86	0.95
	Pool-2 subcohort	0.51	0.05	0.05	0.95	0.96
	Pool-4 subcohort	0.50	0.06	0.05	0.97	0.97
	Synthetic data	0.48	0.05	0.06	0.60	0.83
Censoring	=50%
$\beta_{1}$ :	Individual data	−1.51	0.10	0.09	0.95	0.96
	Pool-2 subcohort	−1.51	0.12	0.10	0.88	0.94
	Pool-4 subcohort	−1.50	0.12	0.10	0.93	0.95
	Synthetic data	−1.53	0.10	0.11	0.78	0.83
$\beta_{2}$ :	Individual data	0.50	0.03	0.02	1.14	0.98
	Pool-2 subcohort	0.50	0.04	0.03	0.97	0.96
	Pool-4 subcohort	0.50	0.04	0.03	1.01	0.93
	Synthetic data	0.50	0.03	0.04	0.82	0.87

NCC: nested case-control; CART: classification and regression trees; HR: hazard ratio; PH: proportional hazards; SE: standard error.

Table 2 shows the estimates obtained for three different event prevalence rates (10%, 30%, and 50%) with each pooled NCC subcohort generated under five controls per case matching. The log HR estimates obtained from the pooled NCC subcohorts are accurate and comparable to the gold standard. However, the SE and bias estimates were generally worse for pooled subcohorts than individual data. We see an inflation of the SE estimates as more samples are pooled from individual to pool sizes 2 and 4, respectively. The confidence interval coverage of the pooled data are also consistent with the nominal 95% coverage of the full cohort. Upon fixing one log HR to zero (HR = 1) and varying the other parameter over the range (−2, 2), the SE estimates obtained for pool-4 were consistently greater than those of pool-2 (shown in Section 1.2 of the Supplemental Appendix). Moreover, the power for a likelihood ratio test (with a type I error rate of 0.05) for the exposure effect (HR, ${\hat{\beta }}_{1}$ in Table 3) shows that the pooled subcohorts are consistently comparable to full cohort data and even outperform all data types when more controls are matched on in rare event settings. Compared to CART data synthesis, the pooled subcohorts consistently produces comparable log HR estimates, less bias, and higher 95% confidence interval coverage. The estimates for matching on two controls are shown in the Supplemental Appendix.

**Table 3.** Power calculations for likelihood ratio tests on ${\hat{\beta }}_{1}$ using 1000 resampled full cohorts with a sample size of 5000. Event prevalence ranged from 5% to 50%, with NCC subcohorts of pool sizes 2, 4, and 10.

$\text{Fix HR}(\beta_{2})=1$				Subcohort pool size		
$\text{HR}(\beta_{1})$	Prevalence	Individual	Synthetic	κ=2	κ=4	κ=10
	50%	0.96	0.95	0.96	0.99	0.99
	30%	0.94	0.94	0.95	0.98	0.99
1.50	12.5%	0.94	0.92	0.94	0.94	0.97
	7.5%	0.95	0.92	0.99	0.99	0.99
	5%	0.97	0.90	0.97	0.98	0.99
	50%	0.81	0.80	0.84	0.86	0.86
	30%	0.80	0.75	0.81	0.85	0.84
1.30	12.5%	0.73	0.96	0.79	0.83	0.88
	7.5%	0.81	0.70	0.82	0.86	0.87
	5%	0.85	0.70	0.85	0.86	0.90
	50%	0.60	0.61	0.61	0.62	0.64
	30%	0.50	0.56	0.57	0.55	0.60
1.10	12.5%	0.51	0.55	0.48	0.52	0.56
	7.5%	0.50	0.50	0.50	0.53	0.60
	5%	0.54	0.48	0.56	0.58	0.68
	50%	0.85	0.86	0.85	0.86	0.88
	30%	0.82	0.81	0.84	0.85	0.84
0.75	12.5%	0.80	0.76	0.82	0.83	0.83
	7.5%	0.83	0.75	0.85	0.86	0.87
	5%	0.83	0.72	0.75	0.86	0.90

HR represents the hazard ratio; NCC: nested case-control.

Finally, pseudo-observed survival times were constructed for the full cohort data using the pooled NCC subcohorts created in Section 2. Mean survival probability plots were then generated for these reconstructions and compared to the exact curve derived from the original full cohort. Figure 4 shows a representative plot of mean survival probability by follow-up time. Overall, our results indicate that the pooled NCC subcohorts are comparable to the full cohort (considered the gold standard) and synthetic data when considering the mean survival probabilities across 1000 simulated samples. Summaries of the median survival time and the restricted mean survival time (a numeric expression of the area under the KM survival curve) are also presented in Table 4. In summary, the proposed reconstructions of pseudo-event times provide a good approximation for the number of individuals comprising the risksets during follow-up.

**Table 4.** KM curve summaries of median survival time ( $T_{1/2}$ ), (2.5,97.5)% credible intervals of $T_{1/2}$ , and the mean values of the restricted mean survival time rmean (SD) for comparing the KM curves of Individual data, pooled subcohorts, and CART-generated synthetic data from 1000 repetitions.

	Individual	Pool-2	Pool-4	Synthetic
Median $T_{1/2}$	0.79	0.75	0.75	0.79
$(2.5,97.5)\%\;T_{1/2}$	(0.73, 0.85)	(0.69, 0.82)	(0.68, 0.82)	(0.71, 0.89)
Mean rmean (SD)	1.10 (0.05)	1.06 (0.05)	1.08 (0.05)	1.11 (0.05)

KM: Kaplan–Meier; CART: classification and regression trees; SD: standard deviation.

**Table 5.** Baseline characteristics of the lung cancer data (full cohort), pooled NCC subcohorts, and synthetic data.

		Dataset			
Variable		Full cohort	Pool-2	Pool-4	Synthetic-Cart
Prevalence	N | died	5,3452 | 1021	3060 | 510	765 | 255	53,452 | 1004
Time (days)	Median (IQR)	2428 (2270, 2553)	2316 (2193, 2436)	2199 (1643, 2313)	2430 (2272, 2554)
Pack years	Mean (SD)	56.0 (23.9)	57.0 (17.5)	59.00 (13.40)	56.2 (24.1)
	Median (IQR)	48.0 (39.0, 66.0)	53.5 (44.0, 66.5)	56.75 (49.0, 66.5)	48.8 (39.0, 67.5)
Age (years)	Mean (SD)	61.4 (5.02)	62.00 (3.74)	62.0 (2.86)	61.4 (5.03)
	Median (IQR)	60.0 (57.0, 65.0)	59.0 (61.5, 64.5)	61.7 (60.0, 64.0)	60.0 (57.0, 65.0)

IQR denotes the interquartile range; SD: standard deviation; NCC: nested case-control.

### 4.2. Lung cancer data example

The National Lung Screening Trial (NLST) lung cancer data^45^ was used to assess the utility of the proposed method. NLST is a randomized controlled trial designed to determine whether screening for lung cancer with low-dose helical computed tomography reduces mortality in high-risk individuals relative to screening with chest radiography. The study enrolled ∼ 54,000 participants, from 33 screening centers across the United States, between August 2002 and April 2004. The participants were followed up until 31 December 2009 when the follow-up period ended. The study contains information on survival of patients (e.g. whether lung cancer was the official cause of death and censored or dead status), days from randomization to death or censored (time in days), patient-reported pack-years, age of the patients, and other comorbidities. For simplicity, we focus on pack-years and age as the exposure and confounder of interest. For the 53,452 patients included in the cohort due to lung cancer, 98.08% were censored. The full cohort data was used to create pooled NCC subcohorts of pool sizes 2 and 4 based on the description given in Section 2. We considered two matched sets of 2 and 5 controls per case. We also generated a synthetic copy of the data using the CART technique introduced earlier. A summary of the baseline characteristics of the NLST data is presented in Table 5.

Cox PH regression was performed to assess the utility of the datasets in characterizing the association between the outcome and the exposure and confounder. The results presented in Table 6 compare the log HR estimates from six data sources: full cohort data of individual level observations (gold standard), Pool-2 and four subcohorts (each of 1:2 and 1:5 matched sets) that combines node-derived pooled data, and synthetic data generated based on the CART data synthesis.

**Table 6.** HR and 95% CI estimates for the associations of pack years and age with lung cancer deaths across the full cohort (individual), pooled NCC subcohorts (1:2 and 1:5 matched sets), and CART synthetic datasets.

Variable	Data source	HR	95% CI
	Individual	1.14	(1.12, 1.16)
	Pool-2 (1:2 NCC)	1.14	(1.11, 1.18)
Pack years	Pool-2 (1:5 NCC)	1.14	(1.11, 1.17)
	Pool-4 (1:2 NCC)	1.15	(1.11, 1.19)
	Pool-4 (1:5 NCC)	1.14	(1.11, 1.17)
	Synthetic	1.13	(1.09, 1.17)
	Individual	1.84	(1.72, 1.96)
	Pool-2 (1:2 NCC)	1.88	(1.69, 2.07)
Age of patient	Pool-2 (1:5 NCC)	1.90	(1.74, 2.05)
	Pool-4(1:2 NCC)	1.77	(1.57, 1.99)
	Pool-4 (1:5 NCC)	1.75	(1.59, 1.91)
	Synthetic	1.63	(1.51, 1.76)

HR: hazard ratio; 95% CI: 95% confidence interval; NCC: nested case-control; CART: classification and regression trees.

Similar to the simulated data results, the HR estimates obtained for the full cohort NLST data are comparable to estimates obtained from the pooled NCC subcohorts and synthetic data. The SE estimates increase as the pool size is increased from 2 to 4 records per pool. As expected with the NCC sampling design, the precision of the parameter estimates also improve as more samples were matched on (e.g. 1:2 versus 1:5 matched case-control sets). For example, the 95% confidence interval of the HR associated with age tightens from (1.69,2.07) to (1.74,2.05) for matching on three additional controls in a subcohort of pool size 2 (i.e. moving from 1:2 to 1:5 matched sets of pool size 2); the confidence interval again decreased from (1.57,1.99) to (1.59,1.91) for the same additional controls when the pool size is 5. A comprehensive summary of all the estimates is presented in Table 6.

## 5. Discussion

In this article, we propose a privacy-preserving analysis technique for time-to-event data within the NCC sampling framework. The technique leverages the pooling scheme introduced by Weinberg and others^16,18,31^ to randomly aggregate individual records across matched sets within the same NCC subcohorts, stratified by the outcome status. Only the aggregated covariate levels of the NCC subcohorts are shared with the analytical site. We employ several data utility metrics to assess the usefulness of the shared aggregate records for Cox PH regression and survival curve estimation under data privacy restrictions. These metrics are applied to the following datasets: full cohort (individual) data, pooled NCC subcohorts with pool sizes of 2 and 4, and CART-generated synthetic data.

The results of our simulations and real data example demonstrate that the HR estimates and SEs obtained through pooled data analysis are comparable to those obtained from individual full cohort records. The estimators derived from the pooled NCC framework are MLEs of the HRs, providing consistent estimates of the individual-level HRs and asymptotically normal results. We also find that matching on more controls during NCC sampling, prior to pooling, leads to a substantial improvement in the efficiency of effect estimates (HRs), as previously reported by Langholz and Goldstein^30^ and Kim.^46^ Bias and relative efficiency estimates of pooled NCC subcohorts are similar to those of individual full cohort Cox regression. The empirical coverage, computed under pooled NCC subcohorts, closely approximates the 95% nominal coverage in most scenarios. Furthermore, survival times reconstructed based on the proposed sampling method are adequate for making meaningful clinical predictions of survival risk using the KM estimator.

The proposed method offers several advantages over competing techniques. For example, matching cases to controls before aggregating the matched sets based on event status is computationally inexpensive and readily accessible via standard statistical software. The communication cost for transferring pooled data across the network is also fairly low, as pooling reduces the overall sample size. The cost efficiency improves as the pool size increases. Moreover, the technique is intuitively simple and accessible to researchers in other fields. In comparison, methods for synthetic data generation are often quite expensive, especially for a large quantity of attributes (e.g. three or more attributes), and might require special and difficult-to-program algorithms. Another noteworthy advantage is that NCC sampling minimizes both selection and recall biases, reducing the risk of re-identification before matched set aggregation. Inclusion of binary effect modifiers and additional confounders is also easily attainable without major modifications to the underlying likelihood.^18^ For example, outcome pooling followed by stratifying by an effect modifier could improve efficiency of estimation. Sometimes, such further stratification is recommended to ensure a fair comparison of measurements. For instance, when the exposures of interest are biomarker measurements from frozen biological material, the cases and controls should be matched based on factors such as storage time or condition. However, the current study recommends random pooling of covariates based on only the outcome status, as the privacy gain in inferential or re-identification risk outweighs the efficiency gain when the patients are further stratified.

Inferential and re-identification disclosure risk are fundamental concerns in assessing privacy. The former is still an active area of research and as such assessing the associated disclosure risk of individuals in the released microdata remains a challenging task.^47–49^ In principle, an intruder (e.g. the analyst or data steward) could infer or learn information about a participant even if exact records were not disclosed. Inferential disclosure risk has been reported to be a major concern in traditional techniques such as suppression of quasi-identifiers or noise perturbation, partly because disclosed records often retain local data structures potentially aiding inference. Pooled NCC subcohorts, in comparison, smooth out underlying local perturbations via summation rendering the risk of inferential disclosure extremely small.^50^

While a comparison of the risk of patient re-identification disclosure of individual records is not covered in the current manuscript, we believe the risk is low in NCC subcohort data for several reasons:^51^ (1) under the generous assumption that all attributes in the database are quasi-identifiers, pooled NCC subcohort data is non-overlapping with individual records of external data sources because aggregates do not retain underlying structures of individual elements, (2) any unique combination of pooled covariates records cannot be an identifying class of the individual components due to random pooling, and (3) any pattern of population overlap between the sensitive individual record and an external source is destroyed via pooling.^50,52^ Furthermore, the distribution of any single aggregate attribute is not an accurate indicator of re-identification disclosure risk, or in the case of a categorical variable the results may be falsely reassuring. In the instances the combination of aggregate attributes leads to a unique combination, individuals remain protected as the aggregates are non-overlapping with external databases of individual records.

Our method has some limitations. First, data aggregation decreases statistical power, potentially reducing the utility of pooled NCC subcohorts compared to individual full cohort data. Consequently, meaningful data patterns could be distorted or completely destroyed via aggregation. Second, selecting a suitable pool size requires the analyst to strike a balance between privacy and utility. While a pool size of 4 has been shown to offer good privacy protection and data utility, even with small to moderate samples,^36,28^ it may be helpful for individual data nodes to assess various pool sizes before sharing the pooled data. For instance, each node could perform a pool-level assessment and share data when the estimates from the largest pool size are comparable to the site’s individual-level estimate. Third, another limitation of publishing pooled NCC subcohort data is the lack of a provable privacy guarantee for the randomized summation mechanism used to generate pools. In recent years, synthetic data generation methods with differential privacy guarantees^53^ have gain popularity. Various classical and modern (deep generative) approaches have since been developed to generate differentially private synthetic datasets. In the future, the development of a pooling mechanism with a mathematically provable privacy guarantee warrants exploration. Lastly, the current method cannot be implemented in heterogeneous data settings where the data nodes harbor different distributions^54^ or with vertically partitioned databases where the attributes are split among the data nodes. In the former case, our approach assumes that the datasets stored at the nodes follow the same distributions and that site-specific subcohorts can be combined without taking between-node differences into account.^55^ A potential modification to account for these differences might be to introduce a weighted likelihood function for evaluating the pooled subcohorts. The latter case remains an active area of research.

Despite these limitations, the trade-off between disclosure risk and utility of pooled disclosed data makes the technique appealing. Pooling individual records at contributing data sites result in minimal utility loss whilst preserving the confidentiality of patients. The additional efficiency gain when more controls are matched-on makes the technique particularly desirable, especially in rare disease settings or in emerging infectious disease studies where the outcome prevalence is still low. In conclusion, the proposed pooling technique of survival time data under NCC subcohort sampling preserves patient privacy while ensuring consistent estimation of effects, suitable standard errors, and accurate survival curves comparable to individual full cohort data.

## Supplemental Material

sj-pdf-1-smm-10.1177_09622802231215804 - Supplemental material for Privacy-preserving analysis of time-to-event data under nested case-control sampling Supplemental material, sj-pdf-1-smm-10.1177_09622802231215804 for Privacy-preserving analysis of time-to-event data under nested case-control sampling by Lamin Juwara, Yi Archer Yang, Ana M Velly and Paramita Saha-Chaudhuri in Statistical Methods in Medical Research
