# Supplementary material for: Privacy-preserving analysis of time-to-event data under nested case-control sampling
Source: Stat Methods Med Res. 2023 Dec 13;33(1):96–111. doi: 10.1177/09622802231215804 (PMC10863373; doi:10.1177/09622802231215804)
Supplement: sj-pdf-1-smm-10.1177_09622802231215804 - Supplemental material for Privacy-preserving analysis of time-to-event data under nested case-control sampling [file sj-pdf-1-smm-10.1177_09622802231215804.pdf]

Appendix for “Privacy-preserving analysis of time-to-event data under nested case-control sampling”

September 24, 2023

1 Simulated data

Consider a survival dataset with a total size of $n = 5000$. Assume the proportional hazards (PH) model $\lambda(t|X) = \lambda_0(t)\exp(\beta_1 X_1 + \beta_2 X_2)$, where the covariates $\mathbf{X} = (X_1, X_2)^T$ are generated from a bivariate normal distribution given by:

$$\begin{bmatrix} X_1 \\ X_2 \end{bmatrix} = \mathcal{N}\left(\begin{bmatrix} 1.5 \\ 2.8 \end{bmatrix}, \begin{bmatrix} 0.04 & -0.024 \\ -0.024 & 0.36 \end{bmatrix}\right) \equiv \mathcal{N}_2(\boldsymbol{\mu}, \boldsymbol{\Sigma}),$$

Where the correlation $\rho_{X_1,X_2} = -0.2$. The regression parameters (β_1, β_2) are set as $(-1.5, 0.5)$. The true failure times T and censoring times C are generated from Weibull distributions with scale parameters $\lambda_k \exp(-\beta_1 X_1 - \beta_2 X_2)$ and λ_C, respectively, while the shape parameter is fixed at 1 for both times. The observed survival time is determined as the minimum of T and C. The values of the fixed parameters λ_k and λ_C are adjusted to achieve varying observed event or censoring prevalence.

1.1 Simulation results: 1:2 nested case-control matched sets

We generate pooled NCC subcohorts using a 1:2 NCC matching for three different event prevalence rates (10%, 30%, and 50%). We assess the same Cox PH model on the full cohort data (Individual), the pooled NCC subcohorts, and synthetic data generated by CART. We present results for the Log HR estimates ($\widehat{\boldsymbol{\beta}}$), the standard error (SE), mean absolute bias (Bias), relative efficiency (Reff), and coverage probability (with a nominal coverage probability set at 0.95).

		Estimate	SE	Bias	Reff	Coverage
Censoring = 10%						
β_1:	Individual data	-1.50	0.13	0.11	0.87	0.94
	Pool-2 subcohort	-1.51	0.14	0.12	0.73	0.86
	Pool-4 subcohort	-1.50	0.12	0.10	0.73	0.89
	Synthetic data	-1.49	0.10	0.13	0.65	0.82
β_2:	Individual data	0.50	0.04	0.03	1.05	0.95
	Pool-2 subcohort	0.50	0.04	0.04	0.92	0.94
	Pool-4 subcohort	0.50	0.04	0.03	0.90	0.93
	Synthetic data	0.50	0.03	0.04	0.80	0.89
Censoring = 30%						
β_1:	Individual data	-1.50	0.14	0.12	0.90	0.95
	Pool-2 subcohort	-1.48	0.19	0.16	0.85	0.93
	Pool-4 subcohort	-1.48	0.17	0.14	0.94	0.93
	Synthetic data	-1.48	0.18	0.17	0.66	0.82
β_2:	Individual data	0.50	0.05	0.04	0.93	0.96
	Pool-2 subcohort	0.51	0.06	0.06	0.88	0.95
	Pool-4 subcohort	0.50	0.06	0.04	1.14	0.97
	Synthetic data	0.51	0.06	0.06	0.72	0.81
Censoring = 50%						
β_1:	Individual data	-1.51	0.13	0.09	1.10	0.97
	Pool-2 subcohort	-1.52	0.14	0.12	0.89	0.96
	Pool-4 subcohort	-1.52	0.12	0.10	0.95	0.94
	Synthetic data	-1.51	0.14	0.17	0.66	0.80
β_2:	Individual data	0.51	0.04	0.03	0.95	0.95
	Pool-2 subcohort	0.50	0.05	0.04	1.04	0.97
	Pool-4 subcohort	0.51	0.04	0.03	0.97	0.94
	Synthetic data	0.48	0.05	0.06	0.60	0.78

Table 1: Log HR ($\widehat{\boldsymbol{\beta}}$) estimates of individual, pooled NCC subcohorts, and CART-generated synthetic data under the Cox PH model assumption. Estimates of standard error (SE), mean absolute bias (Bias), relative efficiency (Reff), and coverage probability are shown. The pools were formed under 1:2 matched NCC subcohorts. Nominal coverage was 0.95.

1.2 Simulation results: 1:5 nested case-control matched sets

1.2.1 Distributions of the Pooled subcohorts *vs* Synthetic data

We present the distributions of the simulated covariates $X_i,\ i = 1, 2$. The NCC subcohorts were created by selecting all the cases and 5 controls per case.

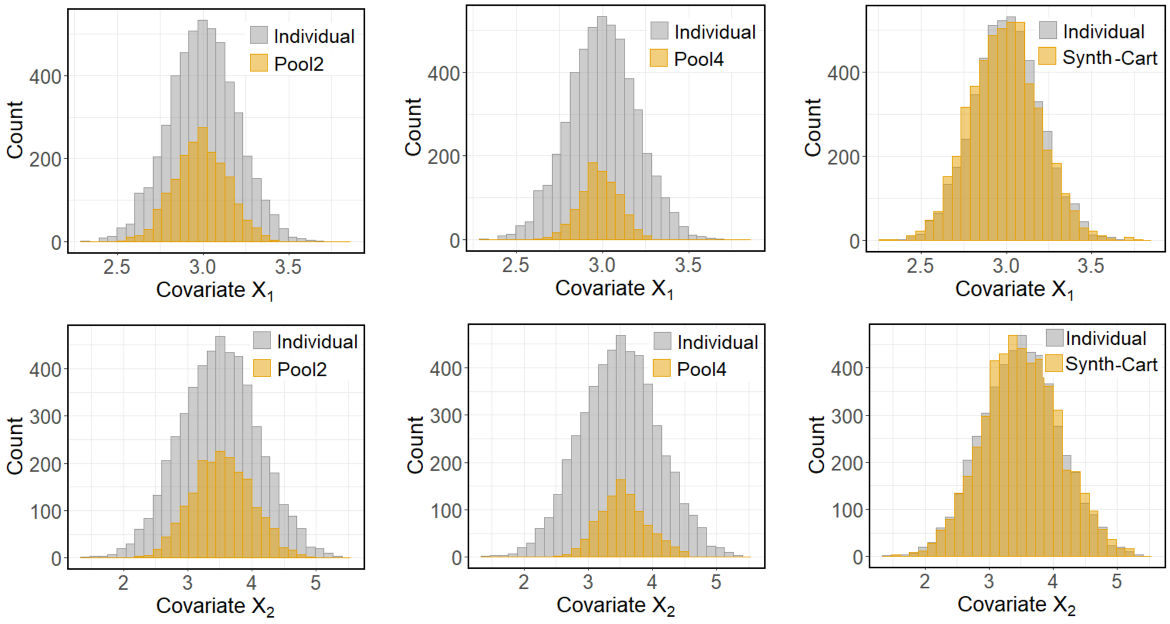

Figure 1: Histogram of pooled NCC subcohorts (of 5 controls per case matched sets) and CART synthetic data overlaid on top of the original dataset. Specifically, Pools of size 2, 4 and synthetic data were plotted on top the full cohort.

1.2.2 Mean Absolute Bias and Standard Error Estimation for 1:5 case-control matched sets

For a more plausible real-life hazard ratio range (e.g., HR estimates between 0.5 and 2), the pooled NCC subcohorts exhibit practically identical estimated standard errors (SEs) to both the full cohort and synthetic data. The SE values remain generally comparable across the entire range of simulated log hazard ratios. Moreover, we observe a sharp increase in the estimated SE when $\beta_1 \to -2, \beta_2 = 0$ due to the asymmetry introduced by taking the exponential of β_1. Conversely, a similar increase occurs when $\beta_2 \to -2, \beta_1 = 0$.

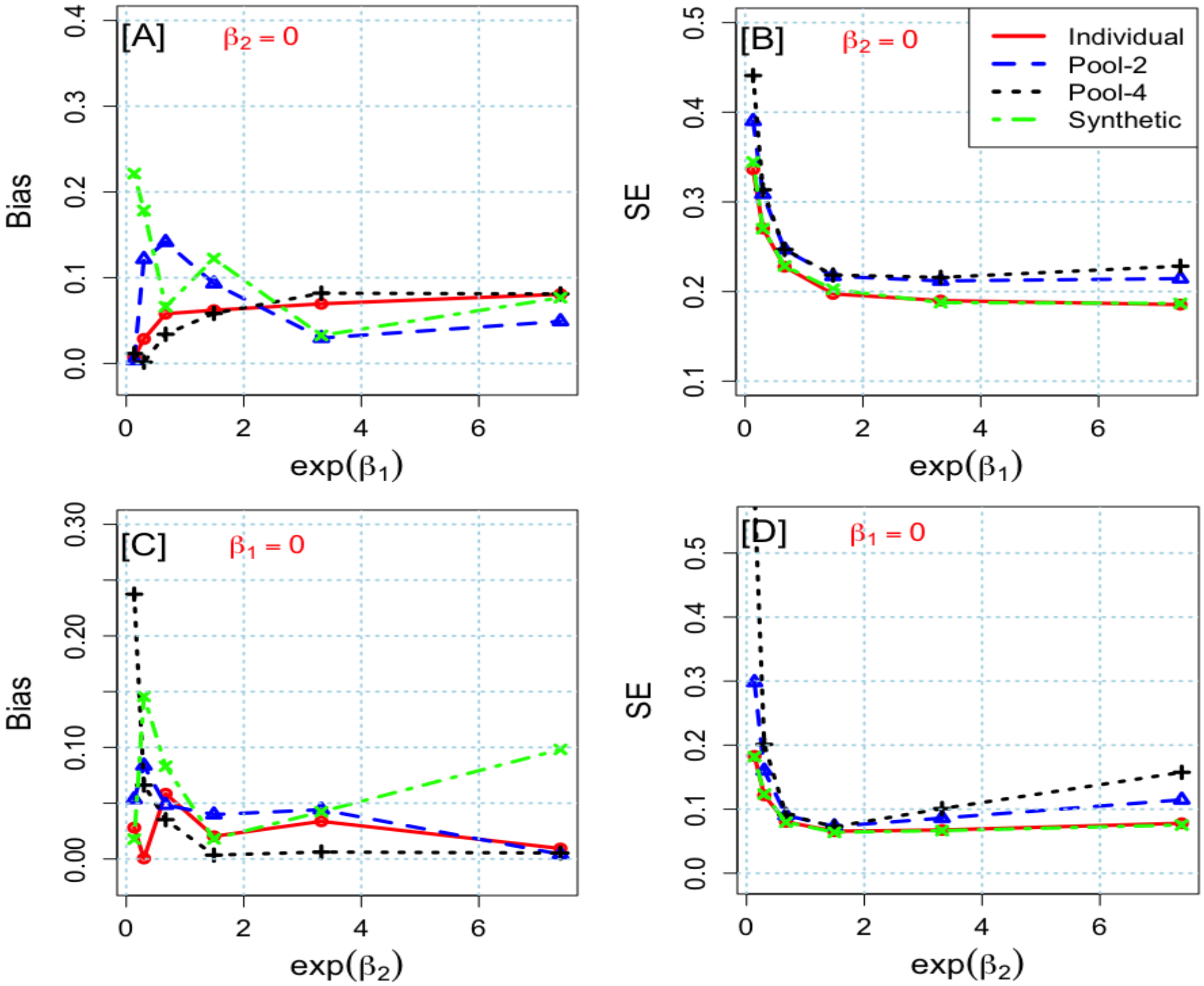

Figure 2: Mean Absolute Bias and Standard Error Estimation: Cox PH Model Applied to Individual Cohorts, Pooled Subcohorts (Size 2 and 4), and CART Synthetic Datasets. The Estimates are Computed over 1000 Simulated Datasets, Each of Size n=5000, for Six Equally Spaced Log Hazard Ratio (log HR) Values in the Interval (-2, 2). Plots are in Terms of Hazard Ratios. The pools were formed under 1:5 matched NCC subcohorts.

1.2.3 Kaplan-Meier survival curves

We reconstructed the survival curve using a randomly selected simulated dataset and present plots for the full cohort (Individual), Pool-2 subcohort, Pool-4 subcohort, and synthetic data generated using CART.

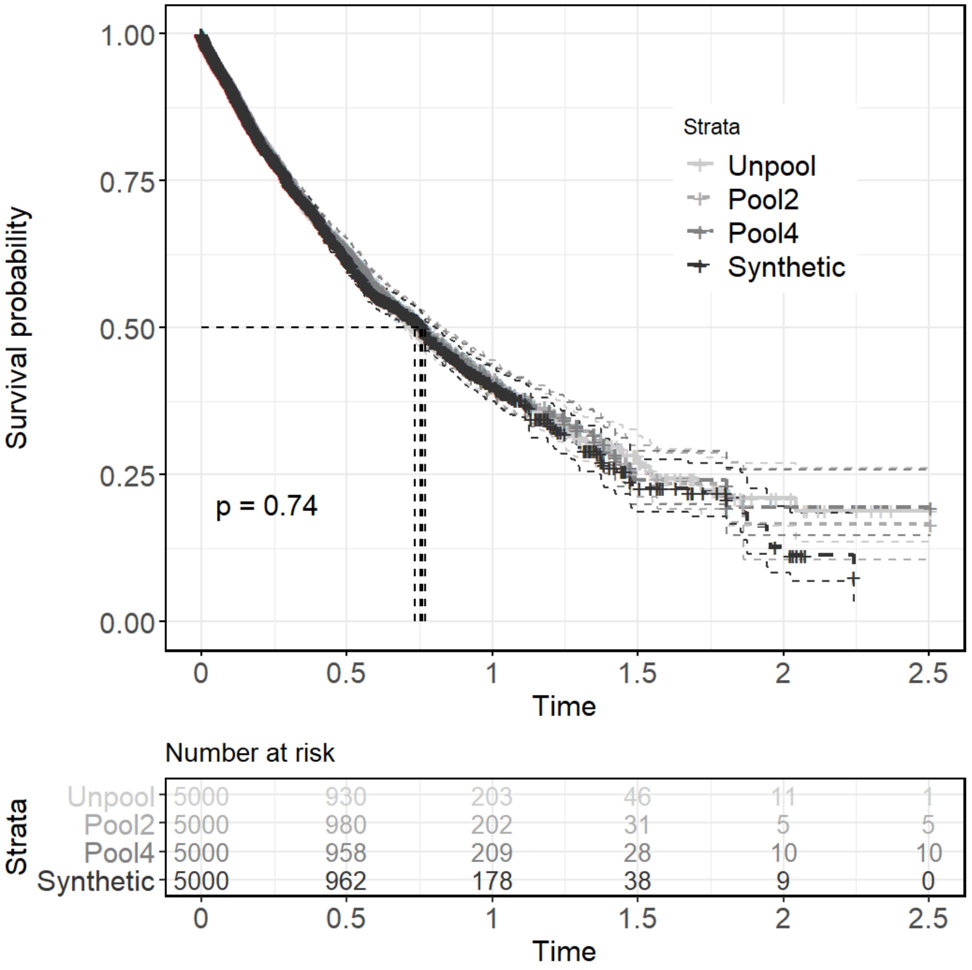

Figure 3: Comparison of Kaplan-Meier Survival Curves: Full Cohort (Unpool), Reconstructed Pooled Subcohorts, and Synthetic Data Generated by CART from a Randomly Sampled Simulated Dataset. The pools were formed under 1:5 matched NCC subcohorts.

2 Classification And Regression Trees for Synthetic Data Generation

Classification And Regression Trees (CART) is a modeling technique that recursively splits the dataset into subsets with more homogeneous outcomes (see Breiman et al., 1984). The splits in the explanatory variable space are typically represented by a tree structure. In Figure 4, we illustrate a tree structure for a univariate outcome Y and two predictors, X_1 and X_2, which was grown using the algorithms proposed by Clark and Pregibon in 1992. The values within each of the final groups (leaves L_1-L_3) approximate a conditional distribution of the predicted variable when the criteria governing that group are met by the predictors. Synthetic data copies are subsequently sampled from these groups. In this manuscript, synthetic data were generated using the *synthpop* package in R.

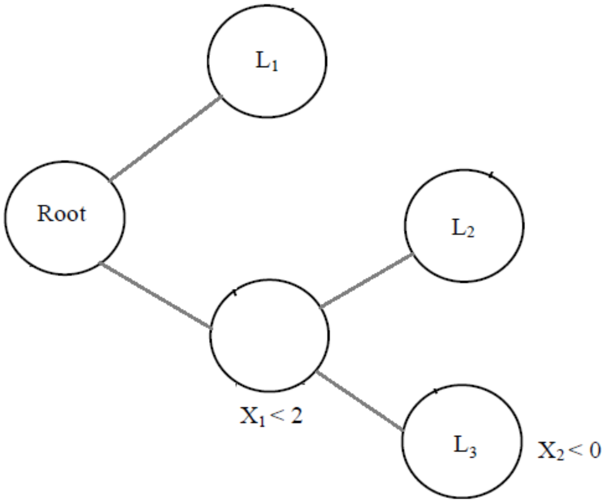

Figure 4: Hypothetical tree structure involving a single outcome and two continuous predictors.
